# Prevalence and Determinants of Intestinal Parasitic Infections among Pregnant Women Receiving Antenatal Care in Kasoa Polyclinic, Ghana

**DOI:** 10.1155/2020/9315025

**Published:** 2020-09-08

**Authors:** Albert Abaka-Yawson, Solomon Quarshie Sosu, Precious Kwablah Kwadzokpui, Salomey Afari, Samuel Adusei, John Arko-Mensah

**Affiliations:** ^1^Department of Biological, Environmental & Occupational Health Sciences, School of Public Health, University of Ghana, Legon, Ghana; ^2^Department of Medical Laboratory Sciences, School of Allied Health Sciences, University of Health and Allied Sciences, Ho, Ghana; ^3^Department of Pharmaceutics, School of Pharmacy, University of Health and Allied Sciences, Ho, Ghana; ^4^Department of Obstetrics and Gynaecology, University of Cape Town, Cape Town, South Africa

## Abstract

**Background:**

Intestinal parasitic infections affect pregnant women worldwide. The infection has been implicated in causing life-threatening conditions in both gravid women and their developing foetus. Sub-Saharan Africa is known to harbor the greatest proportion of intestinal parasitic infections largely due to socioeconomic and environmental factors. In Kasoa, Southern Ghana, there is paucity of data on the prevalence and associated factors of intestinal parasitic infections among pregnant women.

**Objective:**

The aim of the study was to determine the prevalence of intestinal parasitic infections and associated factors among pregnant women attending antenatal care in Kasoa Polyclinic.

**Methods:**

A hospital based analytical cross-sectional study was carried out among three hundred (300) conveniently sampled pregnant women receiving antenatal care services at the Kasoa Polyclinic. Structured questionnaires were administered to the study participants to assess sociodemographic and other possible factors. Stool samples were collected from each pregnant woman and examined for the presence of intestinal parasites by microscopy using direct wet mount as well as formol-ether sedimentation techniques.

**Results:**

Overall prevalence of intestinal parasites was 14.3% (95% CI 11–19%). *Entamoeba histolytica* (5.0%) was the most predominant parasite species identified followed by *Ascaris lumbricoides* (4.3%), *Giardia lamblia* (2.3%), *Trichuris trichiura* (1.3%), *Schistosoma mansoni* (0.3%), Hookworm (0.3%), *Hymenolepis nana* (0.3%), and *Isospora belli* (0.3%). Age > 30 years (AOR = 0.17, 95% CI = 0.06–0.48; *p*=0.001), multigravidity (AOR = 0.43, 95% CI = 0.19–0.97; *p*=0.043), and 2^nd^ and 3^rd^ trimesters (AOR = 4.73, 95% CI = 1.36–16.49; *p*=0.015) were independently associated with intestinal parasitic infections among pregnant women.

**Conclusions:**

A prevalence of 14.3% pregnant women compared to previous studies in Ghana is relatively low. It however suggests that intestinal parasitic infection is still a problem. The major factors noted were age, gravidity, and gestational age. Routine stool examination and provision of public health education are recommended to prevent infection of pregnant mothers and their unborn babies.

## 1. Introduction

Intestinal parasitic infections are primarily caused by protozoans and helminthes [1]. They are frequently transmitted via consumption of contaminated food, bathing, and wading through contaminated water as well as spread from person to person through fecal-oral contact. Intestinal parasitic infections are associated with socioeconomic and environmental factors. They are therefore prevalent in areas where there is overcrowding, limited access to clean water, and poor personal hygiene [2, 3].

Pregnant women, especially those in Africa, are at greater risk of intestinal parasitic infections [4]. A recent study in Ethiopia found 70.6% (553/783) pregnant women to be infected with intestinal parasites with helminthes being the predominant species [5]. In Nigeria, it was observed that 18.2% (73/401) pregnant women were living with intestinal parasitic infections [6]. Data from previous studies in Ghana showed that intestinal parasitic infections among pregnant women were 41.2% [7] and 49.6% [8]. Variations in geographical location and hygienic practices have been identified as important contributing factors to prevalence of intestinal parasitic infections in any population.

Infection with intestinal parasites such as hookworms is known to cause anemia in pregnant women [9], contributing to adverse pregnancy outcomes such as low birth weight and impaired milk production. The roundworm, *Ascaris lumbricoides,* infection has also been implicated in diminished food intake and weight loss in pregnant women [3]. Furthermore it has also been reported to affect survival, growth, and cognitive performance of children born to infected mothers [10].

Additionally, the propensity of trophozoites of the protozoan parasite, *Entamoeba histolytica* to invade the intestinal mucosa, the liver, lungs, and blood circulation of the human host leads to abdominal discomfort, pulmonary abscesses, bleeding episodes, and sometimes death if not diagnosed early for timely treatment [11].

This presents a significant public health situation that requires urgent attention. To deal with this appropriately, the Ministry of Health in collaboration with the Ghana Health Service set up the Neglected Tropical Diseases Control Program (NTDCP) with the rationale of alleviating the occurrence of neglected tropical diseases including intestinal parasitic infections in the country to an insignificant level [12]. This study aimed to determine the prevalence and determinants of intestinal parasitic infections among pregnant women attending antenatal care in Kasoa Polyclinic.

## 2. Materials and Methods

### 2.1. Study Design/Site

A hospital based cross-sectional study using convenient sampling was carried out on pregnant women attending routine antenatal care and laboratory department of the Kasoa Polyclinic. Kasoa is the capital of the Awutu Senya East municipality in the Central Region of Ghana. It has a total of about 69,384 people, which represents more than 79 times its population 40 years ago [13]. Kasoa has main regional market and is known to be a city that for some time has been battling with issues of overcrowding, limited access to treated water, and sanitation facilities [14].

### 2.2. Data Collection and Eligibility Criteria

Closed ended structured questionnaires were then administered with the aid of a local language translator to collect data on their sociodemographic characteristics and potential contributing factors for intestinal parasitic infections. Additionally, pregnant women at various stages of pregnancy were recruited at the Kasoa Polyclinic during attendance to antenatal care services. Pregnant women who have been on any form of antihelminthic therapy within the past three weeks were exempted from the study. Clean screw capped plastic stool containers with wide neck were given to the study participants. The stool containers were identified by prelabeling them with unique identification numbers. The study participants were requested to provide about 2 g (a small spatula attached to the stool container) of stool sample within 24 hours and examined using the direct wet mount method as well as the formol-ether concentration technique for the presence of parasites (trophozoites, cysts, ova, and larvae).

### 2.3. Sample Size

The prevalence of parasitic infections among pregnant women was determined from a Ghanaian study by Fuseini et al. [15] to be 23.0%. The prevalence rate from that study was used to calculate the sample size to be 272. A final sample size of 300 study participants was chosen to account for nonresponse illustrated as follows.

The sample size for the study was calculated using Fisher's sampling formula:

(1) $$\begin{array}{c} N\;=\;\frac{Z^{2}P\left(1-P\right)}{D^{2}}, \end{array}$$

where *N* represents the estimated sample size, *Z* represents the constant for 95% confidence interval given as 1.96, *P* represents the average prevalence of intestinal parasitic infections of 23.0% obtained from a study conducted among pregnant women in Kassena-Nankana district of the Northern Region, and *D* represents the percentage margin of error taken as 5%

## 3. Analysis of Stool Samples

### 3.1. Direct Wet Mount Method

About half of the 2 g stool sample was processed using the direct wet mount method by emulsifying the stool in a normal saline. A drop of the emulsified stool sample was then placed on a labelled glass slide and covered with a coverslip. The preparation was first examined under a 10x objective lens and then 40x for identification of parasites under low light intensity.

### 3.2. Formol-Ether Concentration Technique

For formol-ether concentration technique, about one gram (1 g) of each stool sample was well emulsified with 4 ml of 10% formol saline into a 15 ml conical centrifuge tube through wet cheesecloth-type gauze placed over a disposable paper funnel. The mixture was centrifuged at 3000 rpm for five (5) minutes and the supernatant decanted after centrifugation. An additional 4 ml of 10% formol saline and a next four (4) ml of diethyl ether were added to the sediment, mixed adequately, and then subjected to a second round of centrifugation at 3000 rpm for 5 minutes. The supernatant was then discarded and the concentrated sediments resuspended in 10% formol saline. The sediments were examined microscopically under 10x and 40x magnification for the presence of parasitic organisms as described by Cheesbrough [16].

### 3.3. Quality Control

Known positive and negative slides for the various intestinal parasites were tested using the direct wet mount method and formol-ether concentration method as indicated in the laboratory procedure. Samples and slides were declared positive when various stages of the parasites, such as trophozoites, cysts, ova, and larvae, were observed. This was to ensure reliability of the test procedure as well as a refresher course for the researcher. Additionally, two expert parasitologists were employed to assist with the microscopy.

### 3.4. Ethical Considerations

Protocols used for the study were approved by the Ghana Health Services Ethics Review Committee (Protocol number: GHS-ERC: 067/12/17) and permission was also granted by the Kasoa Polyclinic to carry out the study. Informed written consent was sought from the study participants with the objectives and procedures clearly stated in the consent form. Further, the data collected was treated with enough confidentiality during collection, during analysis, and even after analysis. Possibility of withdrawal from the study at any point was made known to the study participant. Additionally, pregnant women who tested positive were referred to physicians for appropriate medical attention.

### 3.5. Data Analysis

Data collected from the questionnaires and results from the laboratory analysis was checked for consistency and correctness and entered into Microsoft Excel software using fit-for-purpose excel form so as to avoid as much as possible entry errors. The compiled data was analyzed using Stata SE version 15 (StataCorp., College Station, TX, USA). Data is presented as frequencies and standard deviations. The magnitude of association between intestinal parasitic infections and potential risk factors was assessed using univariate and multivariate logistic regression and described in terms of crude odds ratio (cOR) and adjusted odds ratio (aOR) at 95% confidence interval. *p* values of <0.05 were considered statistically significant.

## 4. Results

### 4.1. General Characteristics of Study Participants

The study recruited three hundred (300) pregnant women majority (62.0%) of which were aged 21–30 years and the next most predominant category (32.0%) falling within the age bracket of 31–40 years. Fifty-seven (19.0%) of the women had no formal education, whereas 25.0% (75/300) were able to make it up to their primary education level. In the preponderance of the pregnant women, 116 (38.7%) had their junior high school education with smaller proportions attaining their senior high school tertiary education. More than half (76.7%) of the study participants were married, while 68.3% were gainfully employed.

Predominantly, 57.3% and 73.7% of the pregnant women were primigravidae and in their late trimesters of pregnancy, respectively, possessed their own toilet facility 78% (234/300), and practiced hand washing (96.3%) before meals. A good number of the participants also indicated that they prepared their food at home (85.7%) and drank water from treated sources (96.3%). The study recorded majority, 212 (70.7%), of the participants who indicated that they had dewormed for over 6 months at the time of the study (Table 1).

### 4.2. Prevalence of Intestinal Parasitic Infections

Out of the 300 pregnant women whose stool samples were examined by direct wet mount and formol-ether concentration techniques, 43 (14.3%) of them had intestinal parasites. Out of this, 23 (7.7%) participants had intestinal protozoan parasites and 20 (6.7%) were infected by intestinal helminthes.

The predominant protozoan parasite was *Entamoeba histolytica* which accounted for 15 (5.0%) of infections among pregnant women. This was followed by *Giardia lamblia,* 7 (2.3%), and *Isospora belli*, 1 (0.3%).

**Figure 1:**
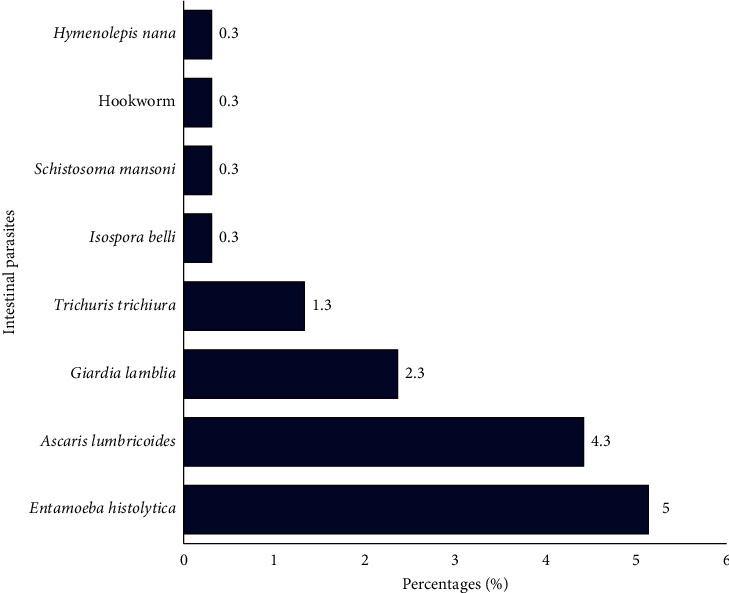
Prevalence of intestinal parasitic infections among pregnant women.

With regard to helminthic infections, the predominant parasite species was *Ascaris lumbricoides* with a prevalence of 13 (4.3%), followed by *Trichuris trichiura* 4 (1.3%). Other parasites seen were *Schistosoma mansoni* 1 (0.3%), *Hymenolepis nana* 1 (0.3%), and Hookworm 1 (0.3%) (Figure 1).

### 4.3. Sociodemographic Factors Associated with Intestinal Parasitic Infections

Table 2 shows the logistic regression analysis of sociodemographic characteristics stratified by intestinal parasitic infections. Three factors were identified to be associated significantly with intestinal parasitic infections by the univariate logistic regression analysis. These include age (cOR = 0.22; 95% CI = 0.83–1.57; *p*=0.002), education (cOR = 0.41; 95% CI = 0.21–0.80; *p*=0.009), and employment status (cOR = 0.38; 95% CI = 0.20–0.73; *p*=0.004).

After adjusting for potential confounders using multivariate logistic regression analysis, only age showed a statistically significant association with intestinal parasitic infections. Pregnant women above 30 years had 83% reduced odds of having intestinal parasitic infections compared to those who were 30 years and below (AOR = 0.17; 95% CI = 0.06–0.48; *p* < 0.001).

### 4.4. Pregnancy-Related and Behavioral Factors Associated with Intestinal Parasitic Infections

In the univariate analysis, factors such as type of toilet facility used, hand washing before meals, and drinking water sources were significantly associated with intestinal parasitic infections among pregnant women. Pregnant women who shared a toilet facility had 2.78-fold greater odds of intestinal parasite infections compared to those who owned a toilet facility (COR = 2.78; 95% CI = 1.40–5.51; *p*=0.004).

Pregnant women who washed their hands before meals had 82% reduced odds of intestinal parasite infections compared to their counterparts who did not (COR = 0.18; 95% CI = 0.05–0.63; *p*=0.007). Also, pregnant women who drank water from untreated sources were 5.5 times likely of contracting intestinal parasitic infections (COR = 5.50; 95% CI = 1.60–18.92; *p*=0.007). Adjusting for potential confounding factors, gravidity was significantly associated with intestinal parasitic infections among pregnant women. There was a 57% reduced odds of intestinal parasitic infections among multigravida women compared to their primigravidae counterparts (AOR = 0.43; 95% CI = 0.19–0.97; *p*=0.043).

Meanwhile, the stage of pregnancy was a significant independent contributor of intestinal parasitic infections among pregnant women. The odds of intestinal parasitic infections among pregnant women in their late trimesters were 4.73 times higher compared to pregnant women in their first trimester (AOR = 4.73; 95% CI = 1.36–16.49; *p*=0.015) (Table 3).

## 5. Discussion

The overall prevalence of intestinal parasitic infections among pregnant women from the study was found to be 14.3% (95% CI 11–19%). This finding is similar to a prevalence of 14.32% obtained from a previous study in Northwest Ethiopia [17], 13.8% in Kenya [3], and 13.0% in Ghana [18]. Relatively higher prevalence rates have been reported in other studies, in Ghana (46.6%) and Ethiopia (70.6%), respectively [5, 8]. In contrast, a study in Iran has reported a significantly lower infection rate of 3.73% [19]. The differences in sociodemographic variables as well as hygienic practices are possible contributing factors to the prevalence of intestinal parasitic infections observed in the above literature.

*Entamoeba histolytica* contributed to the majority of intestinal parasites identified in the study representing a prevalence of 5.0%. This finding differs from that of Forson et al. [20], which recorded a prevalence of 1% and Obiakor-Okeke et al. [21] which recorded no parasites in their study. Though the findings contrast with that of the current study, Akinbo et al. [6] found *E. histolytica* and *Plasmodium* spp. coinfections prevalence to be 16.7% representing the most prevalent. *Entamoeba histolytica* was followed by *Ascaris lumbricoides* (4.3%). The high prevalence of these two parasites (*Entamoeba histolytica* and *Ascaris lumbricoides*) poses a serious health threat to the pregnant women infected. These parasites are known to contribute to bleeding episodes and may lead to adverse pregnancy outcomes that can be life threatening.

With regard to *Giardia lamblia*, its prevalence was found to be 2.3% among the pregnant women. This finding is much higher compared to a prevalence of 1% reported by Akinbo et al. [6] but marginally lower than a prevalence of 2.6% observed by Obiakor-Okeke et al. [21] both in Nigeria.

Hookworm contributed to 0.3% of intestinal parasitic infections identified in the study. This results contrast with that of similar studies which reported higher prevalence of 3.0%, 7.0%, and 38.6%, respectively [6, 7, 22]. The low prevalence rate of hookworm infections observed in this study could be due to good sanitation and proper sewage disposal, as seen in the fact that many participants (78%) had their own toilet facilities rather than shared.

*Trichuris trichiura* accounted for 1.3% of the infections. This was comparable to a prevalence of 1.3% obtained among pregnant women in Kenya [3] but slightly lower than a prevalence of 2.0% observed in Papua New Guinea [23].

Intestinal parasitic infection among pregnant women was determined by age, gravidity, and gestational age. Older age was associated with a reduction in intestinal parasitic infections. This may be due to older women having better knowledge on personal and environmental hygienic practices compared to pregnant women of lower ages. Other studies did not find any association between age and intestinal parasite infections among pregnant women [7, 24, 25].

The stage of pregnancy/gestational age was also found to be associated with intestinal parasitic infections. The late trimesters (second and third trimesters) were associated with increased odds of intestinal parasitic infections among women. This observation is consistent with previous findings where the second and third trimesters of pregnancy presented higher odds of intestinal parasite infections than their counterparts in their first trimester [6, 22]. In contrast to these findings, Espinosa Aranzales and her colleagues have reported that stage of pregnancy had no association with intestinal parasitic infections [25].

Additionally, the study found gravidity to be associated with the odds of intestinal parasitic infections. Multigravida women had reduced odds of intestinal parasitism compared to primigravida women. The findings are in line with that of Phuanukoonnon et al. [23] and Yatich et al. [26]. This observation may be attributed to the fact that multigravida women have previous pregnancy experience and therefore may have benefitted from public health education on various practices to avoid intestinal parasitic infections.

However, none of the sanitation and hygienic practices was associated with intestinal parasitic infections among pregnant women. Whilst the findings are in line with those of Derso et al. [24] and Espinosa Aranzales et al. [25], they are contrary to the findings from Ethiopia, where hand washing practices, availability of toilet facilities, and feeding habits were found to be associated with intestinal parasite infections in pregnancy [5].

A major limitation to this study was the fact that a combination of Kato-Katz method (for helminth species) and formol-ether combination (for protozoa) would give a better estimate of prevalence. Again, PCR would have been more sensitive in the identification and the confirmation of several parasites but financial constraints limited our access to it.

## 6. Conclusion

In conclusion, the overall prevalence of intestinal parasitic infection was 14.3% among pregnant women. The major factors associated with this prevalence were age, gravidity, and gestational age. Therefore, there is need for continuous routine stool examination and health education to prevent infection of pregnant mothers and their unborn babies.

## Figures and Tables

**Table 1:** General characteristics of study participants.

Variable	Frequency	Percentage (%)
Age in years		
≤20	12	4.0
21–30	186	62.0
31–40	96	32.0
41–50	6	2.0

Educational level		
None	57	19.0
Primary	75	25.0
J. H. S.	116	38.7
S. H. S.	38	12.7
Tertiary	14	4.7

Marital status		
Single	70	23.3
Married	230	76.7

Employment status		
Unemployed	95	31.7
Employed	205	68.3

Gravidity		
Primigravidae	172	57.3
Multigravidae	128	42.7

Stage of pregnancy		
First trimester	79	26.3
Late trimesters	221	73.7

Type of toilet facility		
Owned	234	78.0
Shared	66	22.0

Hand washing before meals		
No	11	3.7
Yes	289	96.3

Feeding		
Prepare food at home	257	85.7
Buy food outside	43	14.3

Drinking water sources		
Treated sources	289	96.3
Untreated sources	11	3.7

Deworming history		
≤6 months	88	29.3
>6 months	212	70.7

Data are presented as frequency and percentages; J. H. S.: junior high school; S. H. S.: senior high school.

**Table 2:** Univariate and multivariate analysis of intestinal parasitic infections across sociodemographic characteristics of pregnant women in Kasoa Polyclinic.

Characteristics	Number examined (%)	Number of positive cases (%)	COR (95% CI)	*p* value	AOR (95% CI)	*p* value
Age						
≤30 yrs	198 (66.0)	38 (19.2)	1		1	
>30 yrs	102 (34.0)	5 (4.9)	0.22 (0.83–1.57)	0.002	0.17 (0.06–0.48)	0.001

Education						
Less J. H. S.	132 (44.0)	27 (20.5)	1		1	
J. H. S. and above	168 (56.0)	16 (9.5)	0.41 (0.21–0.80)	0.009	0.55 (0.26–1.18)	0.125

Marital status						
Single	70 (23.3)	9 (12.9)	1		—	
Married	230 (76.7)	34 (14.8)	1.18 (0.53–2.59)	0.688	—	—

Employment						
Unemployed	95 (31.7)	22 (23.2)	1		1	
Employed	205 (68.3)	21 (10.2)	0.38 (0.20–0.73)	0.004	0.49 (0.23–1.05)	0.066

**Table 3:** Univariate and multivariate logistic regression analysis of intestinal parasitic infections with regard to gravidity, deworming status, and hygienic practices.

Characteristics	Number examined (%)	Number of positive cases (%)	cOR (95% CI)	*p* value	aOR (95% CI)	*p* value
Gravidity						
Primigravidae	172 (57.3)	31 (18.0)	1		1	
Multigravidae	128 (42.7)	12 (9.4)	0.47 (0.23–0.96)	0.037	0.43 (0.19–0.97)	0.043

Stage of pregnancy						
First trimester	79 (26.3)	3 (3.8)	1		1	
Late trimesters	221 (73.7)	40 (18.1)	5.60 (1.68–18.65)	0.005	4.73 (1.36–16.49)	0.015

Type of toilet facility						
Owned	234 (78.0)	26 (11.1)	1		1	
Shared	66 (22.0)	17 (25.8)	2.78 (1.40–5.51)	0.004	1.72 (0.77–3.86)	0.186

Hand washing before meals						
No	11 (3.7)	5 (45.5)	1		1	
Yes	289 (96.3)	38 (13.2)	0.18 (0.05–0.63)	0.007	0.25 (0.06–1.03)	0.055

Feeding						
Prepare food at home	257 (85.7)	34 (13.2)	1		—	
Buy food outside	43 (14.3)	9 (20.9)	1.74 (0.77–3.94)	0.187	—	—

Source of drinking water						
Treated sources	289 (96.3)	38 (13.2)	1		1	
Untreated sources	11 (3.7)	5 (45.5)	5.50 (1.60–18.92)	0.007	3.42 (0.87–13.48)	0.079

Deworming history						
≤6 months	88 (29.3)	8 (9.1)	1		—	
>6 months	212 (70.7)	35 (16.5)	1.977 (0.88–4.46)	0.100	—	—

## Data Availability

The data related to this submission are available on request.
